# ComPASSION: A Screening Tool for Type 1 Diabetes and Disordered Eating (T1DE)

**DOI:** 10.1192/bjo.2024.201

**Published:** 2024-08-01

**Authors:** Benjamin Major, Helen Partridge, Sebastian Zaidman, Eveleigh Nicholson, Carla Figueiredo

**Affiliations:** 1 Dorset Healthcare, Bournemouth, United Kingdom; 2 University Hospitals Dorset, Bournemouth, United Kingdom; 3 Portsmouth Hospital University, Portsmouth, United Kingdom

## Abstract

**Aims:**

Background: In 2018, NHS England funded a one-year project of a combined approach for physical and mental health services to support those with type 1 diabetes and eating disorders – ComPASSION Project. Part of this project looked to develop a questionnaire screening tool to improve early recognition of those at risk of T1DE.

Aims: To assess the effectiveness of an adapted questionnaire in identifying patients at risk of T1DE in a routine diabetes clinic. To this end, we focussed on two main aspects:

1. Discussion around weight and body image – patient discussion topic.
2. Diabetes distress score.

**Methods:**

Data from a modified questionnaire was collected retrospectively from diabetes clinics across two hospital sites July 2019–March 2020 with a total study size of 300 patients. Questionnaire responses from those with T1DE were compared with those without.

**Results:**

The questionnaire screening tool is an effective screening tool identifying Type 1 diabetic patients at risk of disordered eating. Patients with T1DE were more likely to raise concerns regarding weight and/or body image. Diabetes distress scores were significantly greater in T1DE patients.

**Conclusion:**

Healthcare professionals should be alert to patients with Type 1 diabetes at risk of disordered eating. Early identification of patients with T1DE is possible when using patient discussion topics and assessing the diabetes distress score. Further studies are needed to assess the effectiveness of this questionnaire screening tool on a larger population.

